# A chronological study of the bacterial pathogen changes in acute neonatal bacterial conjunctivitis in southern China

**DOI:** 10.1186/s12886-017-0570-8

**Published:** 2017-09-26

**Authors:** Song Tang, Ming Li, Hongbo Chen, Guo Ping, Chun Zhang, Shusheng Wang

**Affiliations:** 1 Shenzhen Eye Hospital, Key laboratory of Ophthalmology, Affiliated Shenzhen Eye Hospital of Ji-nan University, Shenzhen, 518000 People’s Republic of China; 2 Department of Cell and Molecular Biology, Tulane University, 2000 Percival Stern Hall, 6400 Freret Street, New Orleans, LA 70118 USA; 3 Department of Ophthalmology, Tulane University, 1430 Tulane Ave, New Orleans, LA 70112 USA

**Keywords:** Neonates, Bacterial conjunctivitis, Pathogens

## Abstract

**Background:**

The aim of the project is to retrospectively study the changes in bacterial pathogens in acute neonatal bacterial conjunctivitis from 2002 to 2016 in Southern China. The results may provide the guidance for drug choice for acute neonatal bacterial conjunctivitis.

**Methods:**

Secretion specimens for bacterial culture were taken from 485 cases with clinically diagnosed acute bacterial neonatal conjunctivitis. Bacterial pathogens were detected by Gram staining and subsequent bacterial culture.

**Results:**

From the analysis of the bacterial pathogens in 485 cases of acute neonatal conjunctivitis patients from 2002 to 2016 in Southern China, there is an overall trend of decreasing detection of Gram-positive bacteria and increasing detection of Gram-negative bacteria from the conjunctival sac secretions. Gram-positive bacteria in the bacteria-positive samples dropped year by year from 82.6% in 2002 to 72.4% in 2016. Accordingly, the ratio of Gram-negative bacteria increased from 17.4% in 2002 to 27.6% in 2016. Of note, despite the overall trend, there was a significant increase in detection of Gram-positive bacteria and decrease in detection of Gram-negative bacteria from 2011 to 2012. Among the Gram-positive bacteria, there is a trend of increasing percentage of the opportunistic pathogens (an ~60% increase in *Staphylococcus epidermidis* and Staphylococcus saprophytic) and decreasing percentage of *Staphylococcus aureus* (~30% decrease) and hemolytic streptococcus (~20% decrease) in the last 15 years. The main Gram-negative bacterium is Neisseria gonorrhoeae. Overall, there is a change in the pattern of bacterial species in acute neonatal bacterial conjunctivitis in Southern China in the last 15 years.

**Conclusion:**

Our study provides a trend analysis of the bacterial pathogens in the conjunctival sac secretions of the acute neonatal bacterial conjunctivitis patients in Southern China in recent years. This data could provide useful information regarding the treatment options for neonatal bacterial conjunctivitis.

## Background

Ophthalmia neonatorum (neonatal conjunctivitis) is the most common eye disease of newborns, which is characterized by redness of the conjunctiva accompanied by of eyelids swelling and purulent discharge [1–7]. Although it is a self-limiting disease, it has the potential of causing blindness if it is not treated timely and correctly. There are multiple etiological factors for the disease, with some septic (bacterial or viral) and some aseptic [8–11]. Bacterial conjunctivitis is the primary cause of blindness in this disease. The pathogen and the drug sensitivity vary across different countries and time periods, which may pose a concern for ophthalmologists and neonatal doctors [8–11]. There are very few reports of chronological studies of bacterial pathogens in the neonatal bacterial conjunctivitis [8–11]. Here we provide the bacterial pathogen and drug sensitivity study of the conjunctival sac secretion specimens from 485 eyes of neonatal patients who were diagnosed with acute neonatal bacterial conjunctivitis in the *Shenzhen Eye Hospital* in Southern China from 2002 to 2016. Our study may provide the rationale for the drug choice in the clinical practice of the disease treatment.

## Methods

### Specimen sources and sample exclusion

Specimens of conjunctival sac secretion were taken from 485 cases of 525 neonatal inpatients and outpatients who were diagnosed with acute neonatal bacterial conjunctivitis in the Shenzhen Eye Hospital and other hospitals in Southern China between 2002 and 2016. The retrospective study was conducted in compliance with informed consent regulations and the Declaration of Helsinki. The study protocol was approved by internal review board (IRB) of Shenzhen Eye Hospital (IRB#: 200,220,111). Informed consent was obtained from the parents of the patients for the study. Patients with lacrimal sac, lacrimal abnormalities and other congenital diseases were excluded. Of the neonatal patients, 265 cases (accounting for 50.5%) were male and 260 cases (accounting for 49.5%) were female, and their ages ranged from 2 to 29 days with a mean age of 8.9 days. The disease was detected in both eyes in 45.9% of the patients (241 cases), whereas 54.1% of the patients (284 cases) were affected in only a single eye. Diagnoses were based on clinical symptoms, including eye redness, epiphora, conjunctival and palpebral inflammation, and serous or purulent discharge.

### Specimen extraction, bacterial culture and identification

Specimens were obtained by gentle application of a sterile swab to the conjunctival surface secretions. The swab did not touch the lashes or eyelid to avoid contamination. The obtained specimen was then spread onto a slide and sent to the laboratory for Giemsa staining and bacterial culture and species identification, and then incubated in a constant-temperature CO_2_ culture incubator at 37 °C. After 48 h, the growth of bacteria was observed, with no bacteria growth as negative, and growers as positive. Bacterial strains were identified through a Delin MicroScan microbial automatic identification system. For samples with bilateral eye involvement, the swaps from both eyes were used for bacterial culture. In these cases, no difference bacterial strains was observed in the left eyes and right eyes.

### Statistical analysis

The data were analyzed using the Statistical Package for the Social Sciences (Windows version 10.0, SPSS Inc.). The data were compared using the χ^2^ test. Linear regression and R square analysis were performed using Microsoft Office Excel (Version 2013).

## Results

*A trend of decreasing Gram-positive bacteria and increasing Gram-negative bacteria in the conjunctival sac secretions from neonatal bacterial conjunctivitis from 2002 to 2016.*

**Fig. 1:**
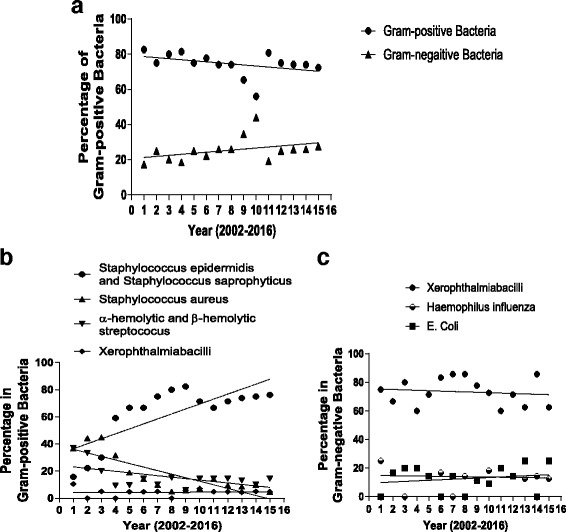
The trends of the Gram-positive and Gram-negative bacteria from patients with neonatal conjunctivitis from 2002 to 2016 in Southern China. **a** Percentage of Gram-positive and Gram negative bacteria from 2002 to 2016. **b** The percentage and trend line of different Gram-positive bacteria identified from 2002 to 2016 responsible for neonatal bacterial conjunctivitis. The percentage of *Staphylococcus epidermidis* and Staphylococcus saprophyticus, *Staphylococcus aureus* α-hemolytic and β-hemolytic streptococcus, and Xerophthalmiabacilli bacteria was shown. **c** The percentage and trend line of different Gram-negative bacteria identified from 2002 to 2016 responsible for neonatal bacterial conjunctivitis. The percentage of Neisseria gonorrhoeae, Haemophilus influenza and *E. coli* bacteria was shown

To detect the bacterial pathogens from neonatal conjunctivitis patients, conjunctival sac secretions were collected and cultured for bacterial detection. From the 485 specimens collected from 2002 to 2016, 400 cases were bacteria-positive, therefore the average positive detection rate is about 83% by the culture (Table 1). There is no detectable trend or difference in the detection rate between the different years (χ^2^ = 0.85, *P* > 0.05). By bacterial culture, 298 of the 400 (74.5%) bacteria-positive specimens were Gram-positive, and 102 of them were Gram-negative (Tables 2 and 3). In all15 years, Gram-positive bacteria were the dominant pathogens presented in the neonatal bacterial conjunctivitis. Interestingly, the proportion of cases with Gram-positive bacteria showed a decreasing trend during the 15-year period. The ratio of Gram-positive bacteria in the bacteria-positive samples dropped year by year from 82.6% in 2002 to 72.4% in 2016 (Linear regression equation: y = −0.6× + 79.3, R^2^ = 0.16, (Fig. 1a)). Accordingly, the ratio of Gram-negative bacteria increased from 17.4% in 2002 to 27.6% in 2016 (Linear regression equation: y = 0.6× + 20.7, R^2^ = 0.16). Of note, despite the overall trend, there was a significant increase in detection of Gram-positive bacteria and decrease in detection of Gram-negative bacteria from 2011 to 2012.

**Table 1:** The rate of positive bacterial detection by culture over the past 15 years in patients with neonatal conjunctivitis

Year	Specimen			Detection rate (%)
	Subtotal	Bacterial positive	Bacterial negative	
2002	29	23	6	79.0%
2003	30	24	6	80.0%
2004	32	25	7	78.0%
2005	35	27	8	77.0%
2006	34	28	6	82.0%
2007	33	27	6	82.0%
2008	32	27	5	84.0%
2009	35	27	8	77.0%
2010	34	26	8	76.0%
2016	31	25	6	81.0%
2012	28	26	2	93.0%
2013	31	28	3	90.0%
2014	36	31	5	86.0%
2015	32	27	5	84.0%
2016	33	29	4	88.0%
Total	485	400	85	Average (83.0%)﻿

**Table 2:** Gram-positive Bacterial identification over the past 15 years in patients with neonatal conjunctivitis

Year	Gram-positive						
	*Staphylococcus epidermidis*	Staphylococcus saprophyticus	*Staphylococcus aureus*	α-hemolytic streptococcus	β-hemolytic streptococcus	Xerophthalmia bacilli	Subtotal (*n*)
2002	2	1	7	3	4	2	19
2003	2	2	8	4	2	0	18
2004	2	4	9	3	1	1	20
2005	6	7	7	1	1	0	22
2006	6	8	4	1	1	1	21
2007	5	9	3	3	0	1	21
2008	8	7	2	1	1	1	20
2009	8	8	1	2	1	0	20
2010	5	9	1	0	1	1	17
2011	8	2	1	1	1	1	14
2012	5	9	3	3	0	1	21
2013	8	7	2	2	1	1	21
2014	8	9	2	2	1	1	23
2015	8	7	2	1	1	1	20
2016	8	8	1	2	1	1	21
Total	89	97	53	29	17	13	298

**Table 3:** Gram-negative Bacterial identification over the past 15 years in patients with neonatal conjunctivitis

Year	Gram-negative			
	Neisseria gonorrhoeae	Haemophilus influenzae	*E. coli*	Subtotal (*n*)
2002	3	1	0	4
2003	4	1	1	6
2004	4	0	1	5
2005	3	1	1	5
2006	5	1	1	7
2007	5	1	0	6
2008	6	0	1	7
2009	6	1	0	7
2010	7	1	1	9
2011	8	2	1	11
2012	3	1	1	5
2013	5	1	1	7
2014	5	1	2	8
2015	6	1	0	7
2016	5	1	2	8
Total	75	14	13	102

### Bacterial identification in the bacteria-positive samples

To identify the common infectious organisms responsible for neonatal bacterial conjunctivitis, eye swabs or conjunctival scrapings were cultured from the 400 bacteria-positive neonatal patients. The most common Gram-positive bacteria were *Staphylococcus epidermis* (*n* = 89), *Staphylococcus saprophyticus* (*n* = 97), *Staphylococcus aureus* (*n* = 53), *alpha-hemolytic streptococcus* (*n* = 29), *beta-hemolytic streptococcus* (*n* = 17), and *Mycobacterium xerosis conjunctiva* (*n* = 13) (Table 2); while the most common Gram-negative bacteria were *Neisseria gonorrhoeae* (*n* = 75), *Haemophilus influenzae* (*n* = 14), and *Escherichia (E). coli* (n = 13) (Table 3).

When the bacterial species in the Gram-positive samples were further categorized, we found *Staphylococcus epidermidis* and *Staphylococcus saprophyticus* accounted for 62.2% (*n* = 186) of the 298 neonatal bacterial conjunctivitis cases, ranging from 15.8% to 82.4% in different years (Table 2). *Staphylococcus aureus* accounted for 17.8% (*n* = 53) of the cases, ranging from 5.0% to 45.0%. *Hemolytic streptococcus* (*alpha- and beta-*) accounted for 15.6% (*n* = 46) of the cases, ranging from 5.9% to 36.8%. *Xerophthalmia bacilli* accounted for 4.5% (*n* = 13) of the cases, ranging from 0% to 10.5%. When the year-by-year data from those 15 years was compared, the proportion of cases involving *Staphylococcus epidermidis* and *Staphylococcus saprophyticus* experienced an increasing trend (increased by ~60%), whereas the proportion of cases with *Staphylococcus aureus* and *Hemolytic streptococcus* (*alpha- and beta-*) showed a decreasing trend in most of the years (decreased by ~30% and ~20% respectively) (Fig. 1b). The linear regression equation of *Staphylococcus epidermidis* and *Staphylococcus saprophyticus*: y = 3.7× + 32.9, R^2^ = 0.59; The linear regression equation of *Staphylococcus aureus*: y = −2.6× + 38.7, R^2^ = 0.67; The linear regression equation of *Hemolytic streptococcus* (alpha- and beta-): y = −1.1× + 24.2, R^2^ = 0.31; The linear regression equation of *Xerophthalmia bacilli* was y = 0.04× + 4.1, R^2^ = 0.0041). However, the proportion of cases with *Mycobacterium xerosis conjunctiva* did not show a clear trend during those years.

When the Gram-negative bacteria in the samples were analyzed, we found that *Neisseria gonorrhoeae* was detected in 73.4% (*n* = 75) of the 102 Gram-negative neonatal bacterial conjunctivitis cases, ranging from 60.0% to 85.7% in different years (Table 3). *Haemophilus influenzae* was detected in 13.7% (n=14) of the cases, ranging from 0% to 25.0%. *E. coli* was detected in 12.7% (n=13) of the cases, ranging from 0% to 25.0%. Although the frequency of the Gram negative bacteria showed an increasing trend during those 15 years, the detection frequency of either *Neisseria gonorrhoeae, Haemophilus influenzae* or *E. coli* seemed to oscillate during the 15 years, without a clear trend identified (Fig. 1c). The linear regression equation of *Neisseria gonorrhoeae*: y = −0.28× + 75.6, R^2^ = 0.017; The linear regression equation of *Haemophilus influenza*: y = −0.11× + 14.9, R^2^ = 0.0054; The linear regression equation of *E. coli* was y = 0.39× + 9.5, R^2^ = 0.037).

## Discussion

To date, few clinical studies have been focused on the long term change of pathogen patterns in neonatal bacterial conjunctivitis. The present study provides a chronological study of the pathogens in acute neonatal bacterial conjunctivitis in Southern China in the past 15 years. We found a trend of decrease in Gram-positive bacteria cases and increase in Gram-negative bacteria cases. Moreover, the change of specific bacterial strains was also analyzed. The knowledge gained from this study could be instrumental for antibiotic drug choice for treating neonatal bacterial conjunctivitis.

### Bacterial pathogen patterns in neonatal bacterial conjunctivitis

Prior research has suggested that *Staphylococcus* and *Streptococcus* were the main pathogens of bacterial conjunctivitis, with *Staphylococcus aureus* and *Streptococcus viridans* identified as the most common pathogens in neonatal bacterial conjunctivitis [1, 2, 12]. According to our research, the pattern of bacterial pathogens in neonatal bacterial conjunctivitis in Southern China has changed during the past 15 years. The number of cases involving Gram-positive bacteria exhibited a decreasing trend, whereas those with Gram-negative bacteria showed a growing trend. In Gram-positive bacteria, the proportion of conditional pathogenic bacteria (*Staphylococcus epidermidis* and *Staphylococcus saprophyticus*) had an increasing trend during the past 15 years, whereas *Staphylococcus aureus* and *Hemolytic streptococcus* had a decreasing trend. These results are consistent with the recent literatures [12, 13]. This pattern change of the bacterial pathogens may be attributed to the combination of neonatal epidermal structure [10, 11], the immature neonatal immune system and the abuse of antibiotics. In the last 15 years, the clinical abuse of antibiotic drugs, especially those against Gram-positive bacteria, may be partly responsible for the decrease of Gram-positive bacteria and increase of Gram-negative bacteria in neonatal bacterial conjunctivitis. On the other hand, the skin and mucus defense system in the neonates are still poor, and the neonatal immune barrier is not fully developed. This lack of defense could make virulent weaker bacteria pathogenic. Therefore, the resident bacteria in the surroundings and opportunistic pathogens (e.g. *Staphylococcus epidermidis*, *Staphylococcus saprophyticus*) may easily cause infection in newborns.

In our study, the two Gram-positive bacteria (*Staphylococcus epidermidis* and *Staphylococcus saprophyticus*) and the Gram-negative *Neisseria gonorrhoeae* account for the majority of the pathogenic bacteria in neonatal acute bacterial conjunctivitis. *Staphylococcus epidermidis* is a type of coagulase-negative bacteria, although it has no pathogenic effect in general. It was detected at high rate on normal skin and conjunctival sac [3, 4]. *Staphylococcus saprophyticus* is another type of coagulase-negative bacteria that has rarely been reported as pathogenic bacteria in conjunctivitis. These two pathogens were previously considered to be opportunistic; however, their existence was increasingly reported recently [5]. Our finding that *Neisseria gonorrhoeae* has become the main Gram-negative pathogen in neonatal acute bacterial conjunctivitis is consistent with an increased incidence of neonatal acute gonococcal conjunctivitis due to the rising incidence of gonorrhea in the last 15 years [6–9].

### Implication in clinical medication of neonatal acute bacterial conjunctivitis

Neonatal acute bacterial conjunctivitis is the most common infectious eye disease in the neonates. Early application of antibiotics is very important in the clinic to control the disease. For severe cases, it is necessary to take samples for bacterial culture and drug sensitivity testing as early as possible before the doctor can modify and find the most efficient treatment [10, 11]. Based on our results, in the clinic, it would be reasonable to consider *Neisseria gonorrhoeae*, *Staphylococcus epidermidis* and *Staphylococcus saprophyticus* first as the pathogens causing neonatal acute bacterial conjunctivitis, if early treatment is necessary. Broad-spectrum antibiotic to both Gram-negative and Gram-positive bacteria, may be considered the first choice of clinical medication to control acute neonatal bacterial conjunctivitis. In this regard, broad-spectrum fluoroquinolone has been shown to be safe and efficient in pediatric population experiencing acute bacterial conjunctivitis [14].

Another implication of the study is that clinical doctors should be cautious about the antibiotics usage in neonatal bacterial conjunctivitis, as well as the consequence of increasing antibiotics abuse in neonatal acute bacterial conjunctivitis in recent years. In the future, attention should be paid not only to the trend of the bacterial pathogen change, but also to the identification of antibiotics resistant bacteria strains.

### Limitation of the study

The strength of this study is the long- term collection of data. However, if the data from each year is considered, the sample size is still small. For example, when the whole data was analyzed, the Gram-negative bacteria tend to increase. However, when the data are separated into each organism and each year, the number is too small for statistical analysis. In this regard, future study with a bigger sample size is warranted.

## Conclusion

Our study provides a trend analysis of the pathogens in acute neonatal bacterial conjunctivitis in South China in the past 15 years, which may help on the clinical decision of antibiotics usage.

## References

[1] Ghosh S, Chatterjee BD, Chakraborty CK (1995). Bacteria in surface infections of neonates. J-Indian-Med-Assoc.

[2] Dannevig L, Straume B, Melby K (1992). Ophthalmia neonatorum in northern Norway. II. Microbiology with emphasis on Chlamydia trachomatis. Acta Ophthalmol Copenh.

[3] Mahajan VM (1983). Acute bacterial infections of the eye: their aetiology and treatment. Br J Ophthalmol.

[4] Seal DV, Barrett SP, McGill JI (1982). Etiology and treatment of acute bacterial infection of the external eye. Br J Opthalmol.

[5] Li, Z., Zheng, J. and Li, J. Diagnostic bacteriology. Hong Kong:Yellow River Culture Press. the first Edition,1992;174–183.

[6] Zanoni D, Isenberg SJ, Apt L (1992). A comparison of silver nitrate with erythromycin for prophylaxis against ophthalmia neonatorum. Clin Pediatr Phila.

[7] Ingram DL (1994). Neisseria gonorrhoeae in children. Pediatr-Ann.

[8] Chen Y (2002). Clinical analysis of 154 cases of neonatal gonococcal conjunctivitis. Acta Universitatis Medicinalis Nanjing.

[9] Zhang S (2000). Analysis of 74 cases of neonatal gonococcal conjunctivitis. Journal of Dermatology and Venereology.

[10] Li M, Lin Y, Chen J (2003). An analysis of bacterial culture in acute neonatal conjunctivitis. Chinese Journal of Practical Ophthalmology.

[11] Li M, Lin Y, Yao X, et al. The vicissitudes of bacterial culture results and antibiotic sensitivity of the pathogenic bacteria in acute neonatal conjunctivitis. Chin Journal of Strabismus & Pediatric Ophthalmology. 2005;13(4):160–4.

[12] Zhang L, Qiu Y, Liu X, et al. Analysis of 59 cases with bacterial keratitis or conjunctivitis which were demonstrated by bacteriology. Chinese Journal of Practical Ophthalmology. 1996;14(11):671–673.

[13] Zhang W, Wu Y, Fan S, et al. A prospective study of maternal—infant transmission of Chlamydia trachomatis. Chinese Journal of Ophthalmology. 1994;30(5):357–358. 7805539

[14] Gross RD, Hoffman RO, Lindsay RN. A comparison of ciprofloxacin and tobramycin in bacterial conjunctivitis in children. Clin Pediatr (Phila). 1997;36(8):435–44. 10.1177/000992289703600801 9272316

